# A novel web-based TinT application and the chronology of the Primate *Alu *retroposon activity

**DOI:** 10.1186/1471-2148-10-376

**Published:** 2010-12-02

**Authors:** Gennady Churakov, Norbert Grundmann, Andrej Kuritzin, Jürgen Brosius, Wojciech Makałowski, Jürgen Schmitz

**Affiliations:** 1 Institute of Experimental Pathology, ZMBE, University of Münster, Von-Esmarch-Str. 56, 48149 Münster, Germany; 2 Institute of Bioinformatics, Faculty of Medicine, University of Münster, Niels Stensen Str. 14, 48149 Münster, Germany; 3 Department of Physics and Mathematics, Saint Petersburg State Institute of Technology, 26 Moskovsky av., St.-Petersburg 198013, Russia

## Abstract

**Background:**

DNA sequences afford access to the evolutionary pathways of life. Particularly mobile elements that constantly co-evolve in genomes encrypt recent and ancient information of their host's history. In mammals there is an extraordinarily abundant activity of mobile elements that occurs in a dynamic succession of active families, subfamilies, types, and subtypes of retroposed elements. The high frequency of retroposons in mammals implies that, by chance, such elements also insert into each other. While inactive elements are no longer able to retropose, active elements retropose by chance into other active and inactive elements. Thousands of such directional, element-in-element insertions are found in present-day genomes. To help analyze these events, we developed a computational algorithm (*T*ranspositions *in T*ranspositions, or TinT) that examines the different frequencies of nested transpositions and reconstructs the chronological order of retroposon activities.

**Results:**

By examining the different frequencies of such nested transpositions, the TinT application reconstructs the chronological order of retroposon activities. We use such activity patterns as a comparative tool to (1) delineate the historical rise and fall of retroposons and their relations to each other, (2) understand the retroposon-induced complexity of recent genomes, and (3) find selective informative homoplasy-free markers of phylogeny. The efficiency of the new application is demonstrated by applying it to dimeric *Alu S*hort *IN*terspersed *E*lements (SINE) to derive a complete chronology of such elements in primates.

**Conclusion:**

The user-friendly, web-based TinT interface presented here affords an easy, automated screening for nested transpositions from genome assemblies or trace data, assembles them in a frequency-matrix, and schematically displays their chronological activity history.

## Background

Discernible transposed elements (TEs) occupy about half of the human genome [1]. They integrate into host DNA in waves of activity. In the face of increasing density, they frequently insert into each other. Nested insertions encrypt valuable historical information about the relative age of the elements, comparable to fossils in distinct layers of earth. As old fossils are absent in young layers, older inactive TEs are not inserted into younger elements. In contrast, young TEs are able to occupy all strata of older elements as well as those active at the same time. Hence TEs active at different historical periods display characteristic insertion profiles. Comprised as they are of a substantial fraction of TEs, mammalian genomes are ideally suited for such analyses. Moreover, even low genomic accumulations (e.g., about 3% genomic coverage of CR1 elements in chicken; [2]), are sufficient for distinct profiles of retroposon activity [3].

Over more than one hundred and sixty million years, mammals have accumulated elements from four major classes of transposons, *L*ong *IN*terspersed *E*lements (LINEs), *S*hort *IN*terspersed *E*lements (SINEs), retrovirus-like *L*ong *T*erminal *R*epeats (LTRs), and DNA transposons [4]. While members of the last group move *via *a cut and paste mechanism, the other three elements transpose by a copy and paste mechanism *via *an RNA intermediate reverse transcribed into cDNA. In humans such RNA transposons represent more than 90% of all transposed elements [1]. Active LINE and LTR elements encode the enzymatic machinery that is necessary for their own propagation, and in the case of LINEs also the co-propagation of SINEs or any other RNA. For LINE1-mediated retroposition, there is a slight preference for A-rich integration sites known as kinkable sites [5]. Such regions contain a TTAAAA consensus motif and are frequently found in the junction of dimeric retroposons such as *Alu *elements in primates. *Alu *elements are primate-specific, 7SL RNA-derived SINEs that arose from Fossil Left and Right *Alu *monomers [6].

A retrospective, sequence-based insight into deep evolutionary periods is feasible *via *inferences from sequence divergence, but is accompanied by uncertainties due to changing regional and temporal substitution rates, mutation saturation, and the occurrence of highly mutated CpG sites. Especially older, highly diverged, and short elements lead to unreliable estimations. Counting and comparing nested insertions, however, is less sensitive to such considerations.

There are currently two different approaches for calculating the relative activity periods of subtypes of transposed elements, both of which draw on RepeatMasker annotations. The *T*ransposon *C*luster *F*inder (TCF) estimates how often certain elements have been fragmented by the insertions of other elements over evolutionary time [7]. A compilation of representative subsets of interacting transposed elements is then presented in an adjacency matrix displaying frequencies of interruptions optimized for their potential chronological order. This *I*nterruptive *M*atrix *A*nalysis (IMA) starts from a random chronological order of elements and systematically repositions them so as to minimize the number of nonzero entries in the part of the matrix defined by the artificial transposition of old elements into new ones.

At about the same time as the TCF application was developed, we developed the *T*ransposition *in T*ransposition (TinT) algorithm [3], which also uses RepeatMasker coordinates to compile interrupted and nested retroposons. The frequencies of fragmented *versus *nested elements are counted, assembled in a data matrix, and sorted by pre-selected retroposon types. This matrix applies a specific probabilistic likelihood model (Additional file 1) to calculate the relative integration period for each retroposon subtype in relation to all other subtypes.

Due to the high frequency and multiple interactions of different elements, both the TCF and the TinT methods exhibit high intrinsic complexities and are neither easy nor self-explanatorily applicable for the scientific community. To compensate for these shortcomings, we have now developed an easy to use, web-based interface for the TinT application. TinTs can be directly screened for in model organisms or in any allocated RepeatMasker report data. To demonstrate and test the web-based TinT method, we investigated the representative primate genomes of *Homo sapiens*, *Macaca mulatta *(rhesus), *Callithrix jacchus *(New World marmoset), *Tarsius syrichta *(Tarsius), and *Microcebus murinus *(grey mouse lemur) and their well-characterized, primate-specific *Alu *dimeric elements. Because of the well-known evolutionary histories of both the species and their retroposons [8,9], primates represent an ideal test group for the TinT application.

## Implementation

The TinT application is implemented in a Java environment (version 1.5 or higher) and executed from a bioinformatics web page that runs as an applet on the client computer. TinT reads and optimizes RepeatMasker information of nested transposons and transfers this information into a data matrix of transpositions in transpositions (TinT). The data matrix is than included in a probability calculation to derive a graphical framework of relative activity periods of transposed elements. The probabilistic model considers a simplified assumption with just one period of activity of elements and no specific target site preference. The applet calculates the relative activity periods of elements, but in the current version no time calibration is implemented. The usage of the web-based application is illustrated in Additional file 2.

## Results

### Principle of the TinT

**Figure 1:**
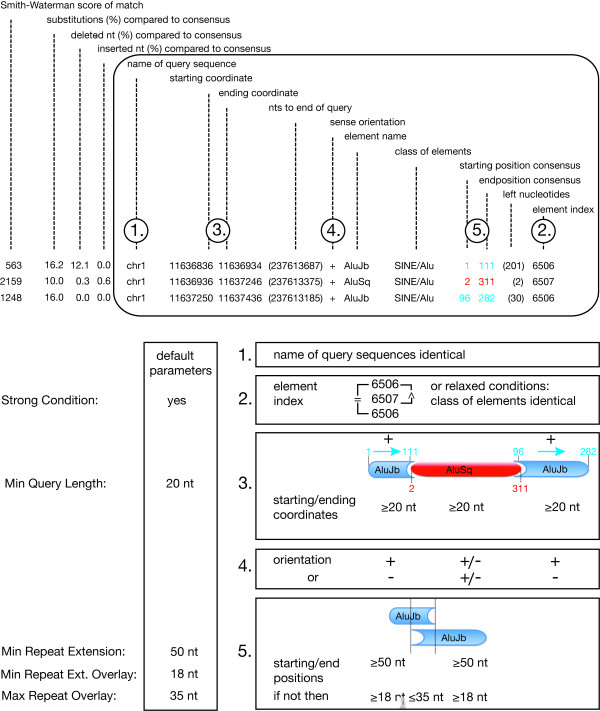
**Interpretation of RepeatMasker outfiles and TinT information**. Schematic representation of the nested insertion of an *Alu*Sq element (coordinates in red) into an *Alu*Jb element (coordinates in blue). The framed area of the RepeatMasker outfile contains the information analyzed by the TinT application and the default parameters. The five characteristics used for extracting unambiguous nested clusters are as follows: (1) fragmented/inserted elements must be located at the same query sequence, (2) for stringent conditions, the element indices for the two parts of the fragmented element must be identical and the index for the nested element must be higher than this; for relaxed conditions the same class of fragmented element parts is sufficient, (3) the minimum size of all elements (starting/ending coordinates) must be 20 nt or more, (4) the fragmented parts of the host element must both be in the same orientation, and (5) the non-overlapping host elements should preferably be larger than 50 nt, but at least ≥ 19 nt with an overlap of no more than 35 nt (starting/end-position consensus).

The first step in generating a TinT profile is to detect nested retroposons. The local version of RepeatMasker http://www.repeatmasker.org/RMDownload.html produces report files containing all necessary information about element types and coordinates of nested and interrupted elements (Figure 1). We considered an element to be unambiguously nested if (1) it is located at the same genomic region as the interrupted element parts, (2) its element index is higher than the identical indices of the interrupted element parts, (3) the starting and end-coordinates of all elements span ≥20 nt each (minimal query length), (4) the interrupted host sequences show the same orientation, and (5) the separated parts of the host element's consensus sequence are preferably ≥50 nt (minimal repeat extension), but at least ≥18 nt (minimal repeat extension overlay) and include an overlap of ≤35 nt (maximal repeat overlay; overlapping host sequence regions are the result of target site duplications or low complexity regions). In cases where the separated host parts have been incorrectly assigned to different subfamilies (as evidenced by detailed retroposon inspection), we adopted the name of the largest part. Single elements (that did not insert or were not fragmented by other elements) were excluded from analyses. Nested integrations of identical elements were used only to tune the parameters of the model. All parameters shown in Figure 1 were optimized by empirical data and can be changed individually. To relax the conditions, the element indexes can be ignored (see Figure 1; element index and Additional file 2: item 4). With this setting, the TinT application only considers whether the interrupted host fragments refer to the same class of elements. Furthermore, the stringency can be altered if the minimal query length, the minimal repeat extension, and minimal repeat extension overlay and/or the maximal repeat overlay is changed. Relaxed conditions are only recommended if the amount of data is reduced or rare elements are involved. If elements are considered that integrate without recognizable target site duplications, such as CR1 elements in birds, the maximal repeat overlay parameter can be reduced and the minimal repeat extension overlay proportionally increased.

After identifying nested retroposons, they were counted, sorted by element subtypes, and compiled in a data matrix. Finally, we developed a symmetric probabilistic likelihood model based on a normal distribution of element activity that transforms the information of the TinT matrix into a pattern of chronological integration periods indicating the probability of activity for each analyzed element type. The underlying mathematical model considers a simple scenario with only one period of activity for each element type and similar probabilities of insertions based on the following assumptions:

1. Elements of type *i *inserted at time points $\tau_{k}^{i}$ (*k *= 1, 2, ..., *n*_*i*_; where *n*_*i *_is the number of all elements of type *i*).

2. In each of such points in time, inserted element of type *i *may fragment some elements of type *j *with a certain probability $p_{i,j}(\tau_{k}^{i})$ (including the case: *j *= *i*).

3. Considering an identical probability of insertion into any preexisting element, denoted by α, then probability $p_{i,j}(\tau_{k}^{i})$ can be represented as $p_{i,j}(\tau_{k}^{i})=\alpha \cdot \eta^{j}(\tau_{k}^{i})$, where *η *^*j *^(*t*) is the number of elements of type *j *preexisting at time point *t*.

4. Function *η *^*j *^(*t*) is approximated using the normal (Gaussian) distribution with mean *t*_*j *_and standard deviation *n*_*j*_, e.g., $\eta^{j}(t)=\frac{n_{j}}{\sqrt{2\pi }}\int_{-\infty }^{\frac{t-t_{j}}{\sigma_{j}}}e^{-\frac{y^{2}}{2}}dy$, where *σ*_*j *_= *n*_*j*_, and its derivative $\frac{d}{dt}\eta^{j}(t)=\frac{1}{\sqrt{2\pi }}e^{-\frac{{\left(t-t_{j}\right)}^{2}}{2n_{j}^{2}}}$ has the maximum at time point *t *= *t*_*j*_. All details of the model are presented as Additional file 1.

### Web-based version of TinT

The web-based version of TinT is located at http://www.bioinformatics.uni-muenster.de/tools/tint and requires Java version 1.5. The application is written as a java applet and was developed using the multi-language software development environment Eclipse, which is an integrated development environment with a repository system (CVS) in the background that keeps software changes disposable. There are two input options. First, any RepeatMasker report file can be uploaded and variable subsets and combinations of elements can be selected for TinT calculations. Furthermore, pre-analyzed model organisms and specific elements can be selected for a TinT analysis. Currently, 19 pre-computed genomes are available for the TinT analysis and the data (RepeatMasker output files) can be downloaded from http://www.bioinformatics.uni-muenster.de/tools/tint/download/RepeatMasker/.DIR. The TinT activity pattern is then graphically displayed. It should be mentioned that the application is executed locally on the computer where it is accessed. Optional parameters for reading RepeatMasker data may be entered into a special dialog box. Transpositions can be grouped and this information can be loaded from a file to provide flexibility for further analysis. Printing or exporting the generated graphs is a basic part of the software, so the results can be used in other applications. Exporting depends on the standard printer dialog of the computer system - if available the print is directed to a postscript file.

### An example data set from primates

**Figure 2:**
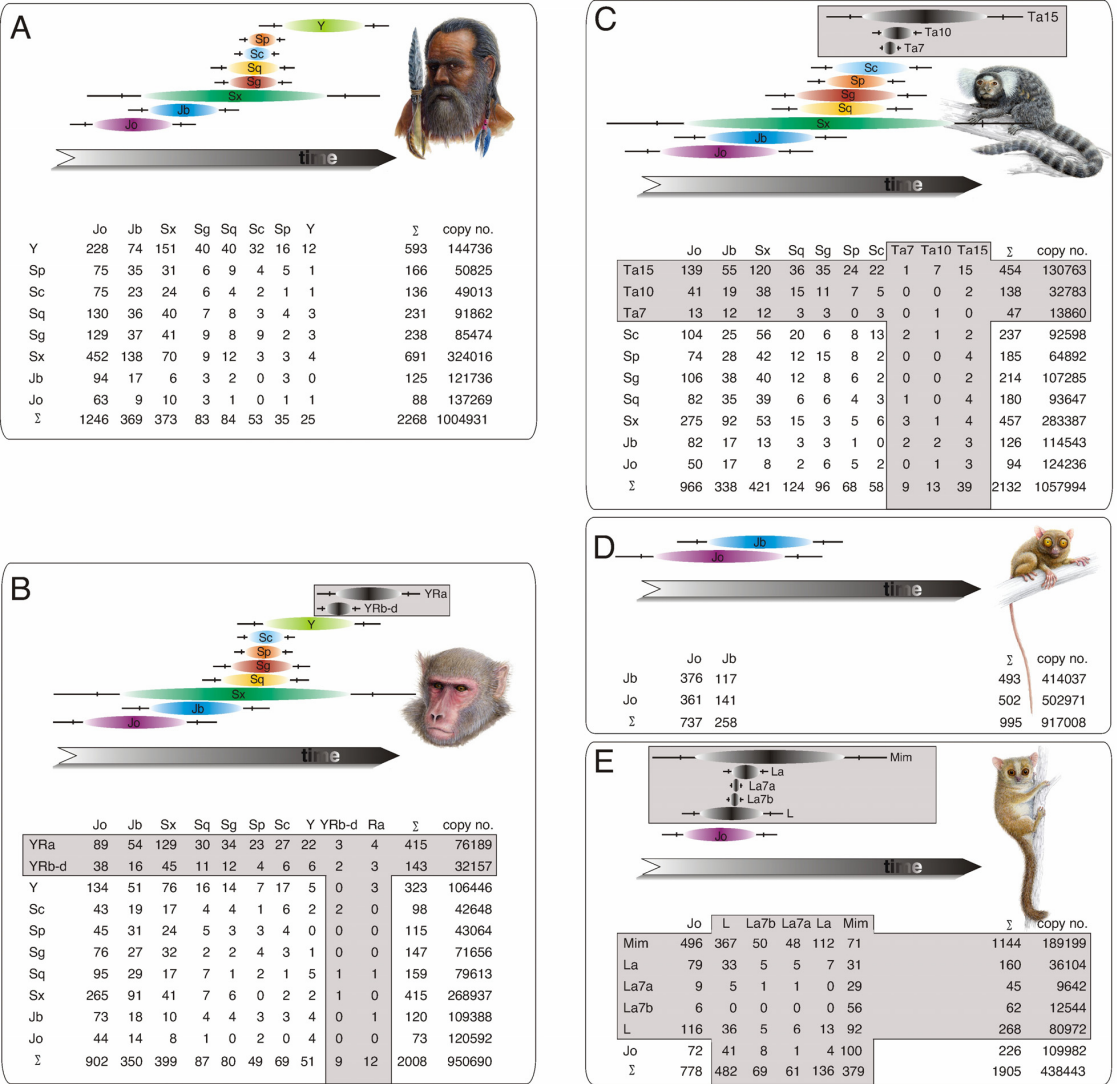
**Results of the TinT analysis for different *Alu *dimers in primates**. The lower part of each panel represents the data matrix derived from the RepeatMasker outfile. Subtypes listed across the top represent host elements; those listed along the left side are the inserted elements. The copy numbers of *Alu *elements are indicated in the last column and the sums of nested insertions for both hosts and inserted elements are shown across the bottom and to the right, respectively. The upper parts of each panel present a graphical display of the chronological activities of the elements sorted by the peak of each activity. The center of each oval represents the maximum of each activity period; the ends of each oval encompass 75%, the vertical lines 95%, and the ends of each line 99% of the probable activity period range. Elements within grey boxes are taxon-specific elements. A relative time scale is shown below. TinT profiles for (A) *Homo sapiens*, (B) *Macaca mulatta*, (C) *Callithrix jacchus*, (D) *Tarsius syrichta*, and (E) *Microcebus murinus*.

After selectively screening the human genome with RepeatMasker, we detected 1,004,931 dimeric *Alu *elements, 2,268 of which were considered to be unambiguous nested insertions. The frequencies of insertions extracted from the retroposon matrix (Figure 2A) were used to calculate their activity probabilities (Additional file 2). Because of the multidimensional insertion pattern, the probable relative activity of each given element subtype is directly interrelated to those of the other subtypes. *Alu*Jo appears as the first active *Alu *dimer, followed by *Alu*Jb. *Alu*Sx shows the most expanded activity with the 75% interval of probable activity overlapping that of both the *Alu*J elements and the other *Alu*S subfamilies. The *Alu*Y elements are clearly separated from the older elements and contain those that are currently still active representatives of *Alu *dimers (Additional file 2).

From the rhesus monkey (*Macaca mulatta*) genome sequences we detected 950,960 dimeric elements including 2,008 nested, TinTs. The pattern of these *Alu *dimers (Figure 2B) is similar to that of human; but includes, in addition, rhesus monkey-specific, *Alu*Y-related *Alu*R elements [10]. Because the resolution of individual *Alu*YR elements was too low, the related *Alu*YRb-d elements (TinT-option: merging elements) were combined.

The 2,132 nested elements from the 1,057,994 *Alu *elements detected in the New World marmoset (Figure 2C) also show a distribution comparable to those of human and rhesus monkey. In addition, there are three New World monkey-specific *Alu*Ta elements with the most recent activities [11].

The 995 nested elements from the 917,008 dimeric *Alu *elements detected in the Tarsius genomic sequences (Figure 2D) comprise only the two known *Alu*J elements. The TinT analysis showed that *Alu*Jo was older than the *Alu*Jb. Tarsius-specific *Alu *elements have not yet been detected.

The RepeatMasker screen of the gray mouse lemur (*Microcebus murinus*) sequences (Figure 2E) revealed a total of 438,443 *Alu *elements, of which 1,905 were unambiguously nested. The mouse lemur lacks the *Alu*Jb elements that are present in all other primate groups. In addition to other potential but as yet uncharacterized *Alu *dimers, there are several recently described, lemur-specific *Alu *elements with very dominant and recent distributions (*Alu*L, *Alu*La, and *Alu*Mim; Repbase; [12]).

### Comparison to the TCF defragmentation pattern

Giordano et al. [7] presented their fragmentation analysis based on a Transposon Cluster Finder (TCF) software package. The transposon defragmentation analysis included most known mammalian TE classes and families but only the three main *Alu *groups *Alu*J, *Alu*S, and *Alu*Y. Therefore a direct comparison to our TinT activity pattern of *Alu *elements is limited. Furthermore, the TCF software is not freely available to derive a comparable set of data. The TCF pattern for *Alu *elements roughly confirms the TinT-derived succession of these elements, but indicates an artificial activity overlay of *Alu*J with *Alu*S and *Alu*Y elements.

## Discussion

In light of the many ongoing genome sequence projects, the TinT method should prove to be quite valuable for characterizing the retroposon-influenced architecture and evolutionary history of genomes and provides a basic aid in conducting efficient retroposon-based phylogenetic reconstructions. To test and demonstrate the advanced efficiency of the TinT algorithm and to present a user-friendly web-based application, we performed a comparative analysis of nested primate specific dimeric *Alu *SINEs, a group of elements with an established evolutionary history [8,9]. Using standard consensus sequences of *Alu *repeats [13] to screen all available genomic sources of primates, represented by human and macaque (both Catarrhini), marmoset (Platyrrhini), Philippine Tarsius (Tarsiiformes), and gray mouse lemur (Strepsirrhini), we extracted and analyzed more than 9,300 nested from 4.5 million detected *Alu *SINEs. The relative activity periods of *Alu *elements revealed by the TinT analyses coincide with our current knowledge of these elements in primates [14].

It should be mentioned that a substantial proportion of the nested elements are ancestral insertion events and consequently are shared among different primate groups. Such common TinTs lead to similar activity patterns of species, especially for older elements (see also for example [15], Figure 1).

**Figure 3:**
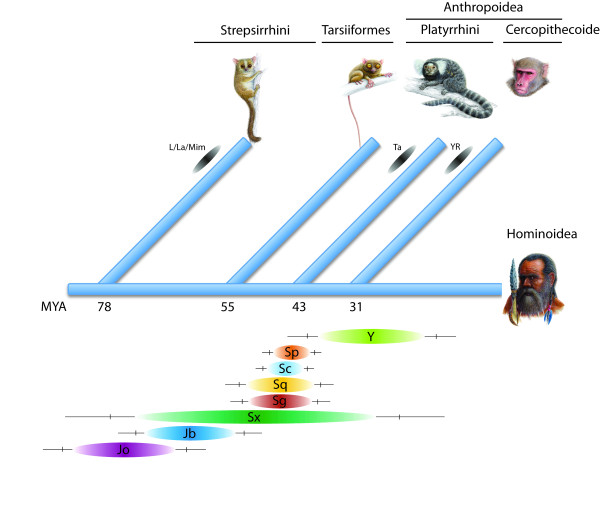
**TinT activity patterns and species evolution**. Schematic representation of the phylogenetic relationships of the five major primate groups: Strepsirrhini (represented by *Microcebus murinus*), Tarsiiformes (represented by *Tarsius syrichta*), Platyrrhini (represented by *Callithrix jacchus*), Cercopithecoidea (represented by *Macaca mulatta*), and Hominoidea (represented by *Homo sapiens*). The dating is taken from [25,26]. *Alu*Jo elements were active at the divergence of Strepsirrhines and *Alu*Jb at the divergence of Tarsiiformes. The main activity of *Alu*S elements occurred around the evolution of Anthropoidea; *Alu*Y elements arose on the lineage leading to Cercopithecoidea and are still active in Catarrhini, including *Macaca mulatta *and *Homo sapiens*. Group-specific *Alu *elements are indicated at terminal branches (L = *Alu*L, La = *Alu*La, Mim = *Alu*Mim; Ta = *Alu*Ta; YR = *Alu*YR).

Nine diagnostic mutations distinguish *Alu*Jo from *Alu*Jb [14]. The TinT profiles support the activity of *Alu*Jo having preceded that of *Alu*Jb and, with the probable absence of *Alu*Jb in strepsirrhines, indicate an origin of latter elements in the common ancestor of Tarsius and higher primates. The phylogenetic affiliation of these two groups in a clade Haplorrhini was previously significantly supported by four orthologous insertions of retroposed elements [16,17]. This relationship is now overwhelmingly supported with quantitative and chronological evidence from 414,037 *Alu*Jb elements specific for Haplorrhini that are clearly absent in strepsirrhines. Beside some few specific elements humans and macaques have nearly identical profiles of *Alu *SINE activity. Similar activity profiles for older *Alu *SINEs (*Alu*Jo, *Alu*Jb and *Alu*Sx) were also detected in New World monkey (marmoset). In contrast, the overlapping activity patterns of the younger *Alu*S and *Alu*Y SINEs vary among primate groups. The TinT patterns of element activities (Figure 2) fit well to the sequence-based reconstruction of the evolution of *Alu *elements (Additional file 3) and to the commonly accepted phylogenetic tree of primates (Figure 3). Three implications can be drawn from the TinT patterns of *Alu *SINEs: (1) several subtypes of *Alu *elements were active during overlapping periods, (2) a significant change in *Alu *activity took place after Tarsius separated from a common ancestor with anthropoids, and (3) the TinT activity profiles correlate perfectly with the well known activity patterns of *Alu *elements [14]. Comparing TinT profiles of dimeric *Alu *elements to the phylogenetic relationships of different primate species documents the correlation between the activity of retroelements and species evolution (Additional file 3).

However, comparing the TinT analysis of dimeric *Alu *elements to the TCF defragmentation pattern [7] demonstrates that the TinT analysis provides a more accurate activity pattern and implements information about the number of elements in the genome. The TCF defragmentation analysis shows an *Alu*J activity time span that overlaps with that of both *Alu*S and *Alu*Y elements. The TinT profiles clearly indicate that *Alu*J elements were already silent before the divergence of Anthropoidea and before the appearance of most *Alu*S subtypes and *Alu*Y (Additional file 3). Furthermore, the TCF analysis requires that any given element type interact with at least 29% of all other analyzed element types. The TinT model requires that a given element type interact with only two additional types. Especially for small amounts of genomic data, this raises the sensitivity drastically.

For TinT analyses it is important to carefully preselect and compile the elements of interest. Large elements (e.g., the 6,000 bp, full length, primate-specific L1P type of LINEs) have a higher chance of being occupied by other elements than do smaller ones (e.g., the 300 bp *Alu *SINE elements.) Therefore, we advise users to analyze such groups separately (see for example [18]). TinT analyses of both monomeric and dimeric elements together should be interpreted with care. *Alu *elements for example are composed of two monomers connected via an oligo(A) sequence. Such A-rich regions are preferred targets for insertions [5,19] and can bias the insertion profile.

## Conclusions

The insertion patterns of retroposed elements provide a homoplasy-free character set for tracing the evolutionary history of species [20]. The insertion of a given element at the same genomic location in two species and its absence in a reference species indicates a close relationship between the two sharing species [21]. However, randomly choosing retroposed elements for laborious phylogenetic analysis is highly inefficient, while preselecting specific informative element types (e.g., for deep phylogenetic splits) significantly raises the efficiency of downstream experimental analyses. The TinT application provides *a priori *information about the relative activity periods of given elements (e.g., to investigate old splits by selectively analyzing old elements that were active in the potential common ancestor of a specific group). The phylogenetic application of TinT-derived element activities significantly aided in resolving the evolutionary histories of galliformes [3], marsupials [15], and lagomorphs [22].

It is well known that retroposed elements significantly influence genome evolution, architecture, and gene function; hence, a clear understanding of their insertion events is a key to understanding the genomic architecture of present-day genomes. Therefore, in addition to a statistical compilation of such elements, TinT affords an invaluable tool for analyzing the chronological activity of retroposed elements. Because SINE elements depend on the LINE retroposition machinery for their insertion, their activity is closely connected to LINE activity. As an example, mammalian-wide interspersed elements (MIRs [23]) coincide with the activity of LINE2 elements and presumably the propagation of MIRs ended after the inactivation of such elements. To understand the dependence of SINE-LINE associations, overlapping activity periods are a first indication of potential interactivity. A potential non-autonomous and autonomous element affiliation was shown for a novel SINE-like snoRTE element and BovB_Plat autonomous retroposons in platypus [24].

Genome-wide chronological analyses of transposed elements using TinT build on the RepeatMasker detection of elements or fragments thereof. The detection is based on sequence similarity to a predefined compilation of transposons. Although TinT performs a subsequent stringent quality-check of detected fragmented elements, miss-annotations, especially if old and thus highly diverged elements are involved, cannot be completely excluded. Therefore, more sequence data leads to an increase in precision.

In future TinT updates, we plan to implement two additional levels of complexity. By a genome-wide pre-screening of element-specific insertions, we intend to add empirical retroposon information of type-specific (monomer-dimer, short-long elements) insertion probabilities; thus, freeing the algorithm from the assumption that all elements have similar insertion probabilities. Associated with this, we intend to improve the accuracy of TinT analyses by introducing an asymmetric model of element activity, whereby elements will not necessary reach their highest probability of activity at the center of their activity range. Furthermore, we plan to incorporate an absolute time scale of activity by incorporating divergence data of elements.

## Methods

### Required RepeatMasker Input Data

The RepeatMasker source file can be a critical source of errors due to miss-annotations of elements or their fragments. To overcome this potential problem, we developed a quality check of the RepeatMasker TinT coordinates and automatically selected only unambiguously nested insertions for our analyses. The stringent selection works well for genome data and frequently occurring elements, and provides a reliable TinT pattern. For the analysis of lesser quantities of data or genomes with low copy number elements, we have provided the option of applying less stringent parameters (relaxed conditions; Figure 1).

However, for the human genome, the minimal amount of data that is necessary under stringent conditions to retain a full TinT resolution is about 10% of the genome, for instance about 300,000 traces are sufficient to receive the representative full TinT pattern. This means, the pattern is stable and reproducible after adding additional portions of data. It is noteworthy that this calculation varies from species to species and depends on the frequency of available elements. The precision of the TinT approach increases with the amount and quality of the input data. To derive the most reliable TinT pattern, all available sequences of selected species should be downloaded from genome (ftp://ftp.ncbi.nih.gov/genomes/ or trace databases ftp://ftp.ncbi.nih.gov/pub/TraceDB/). The most time-consuming step of the TinT analysis is the upstream RepeatMasker screening. Depending on the amount of genomic data, the size of the RepeatMasker library used, and the available computational power, this process might run for several days. To reduce this screening time, it is advisable to restrict the RepeatMasker library to specific element groups (e.g., SINEs or LINEs). The local RepeatMasker library can be assembled with specific elements or element groups. Similar retroposon types should always be included in one run to avoid artificial annotation of the masked repeats. The report file can be directly applied to downstream processes.

### Primate Test Sets of Data

Genomes of *Homo sapiens *(hg19), *Macaca mulatta *(rheMac2), and *Callithrix jacchus *(calJac1) were downloaded from the UCSC Genome Bioinformatics site; http://hgdownload.cse.ucsc.edu/downloads.html; *Tarsius syrichta *(Tarsyr1.0) and *Microcebus murinus *(MicMur1.0) genomes were downloaded from the Broad Institute; http://www.broadinstitute.org/ftp/pub/assemblies/mammals.

## Availability and Requirements

Project name: TinT

**Project home page**: http://www.bioinformatics.uni-muenster.de/tools/tint

**Operating system**: Platform independent (Requires a Java Virtual Machine (JVM) on the target system)

**Programming language**: Java

**Requirements**: Java Runtime Environment

**License**: GPL for academic users

## Authors' contributions

GC, JS, WM, and NG conceived of the user-friendly TinT web-interface. AK and GC adjusted the probabilistic model of TinT, NG and WM implemented TinT into the Java-based web-interface. JB contributed computational equipment. JS wrote the paper. All authors read, edited, and approved the final manuscript.

## Supplementary Material

Additional file 1 TinT probabilistic model Click here for file

Additional file 2 TinT tutorial Click here for file

Additional file 3 Sequence-based phylogeny of *Alu *elements Click here for file
