# Supplementary material for: A novel web-based TinT application and the chronology of the Primate Alu retroposon activity
Source: BMC Evol Biol. 2010 Dec 2;10:376. doi: 10.1186/1471-2148-10-376 (PMC3014933; doi:10.1186/1471-2148-10-376)
Supplement: Additional file 1 — TinT probabilistic model [file 1471-2148-10-376-S1.PDF]

Additional file 1: **TinT probabilistic model**

The following derivation is based on a series of N independent tests. Each test can have $N+1$ results. The number of results can be from 0 up to N. However, if any of the results at special time points are impossible, we consider their probabilities equal to 0.

We designate $p_{i,j}(t)$ as the probability of the results j for experiments of the i-th series at time point t (i and $j \in \{1,2...N\}$, $t \in T_i = \{\tau_1^i, \tau_2^i, ..., \tau_{n_j}^i\}$). T_i is the set of time points in which the tests are performed.

We consider, that for any $t \in T_i$ $\sum_{j=0}^{N} p_{i,j}(t) = 1$.

We assume then that $p_{i,j}(t) << 1/N$ (accordingly, $p_{i,i}(t)$ is close to 1).

In the experiment, values $m_{i,j}$ of the random variables $\mu_{i,j}$ are the numbers of approaches of results j in the i-th series under the condition that all combinations i and $j \in$ $\{1,2...N\}$.

We consider $n_i = \sum_{j=0}^{N} m_{i,j}$ as the number of tests in the i-th series.

We define $\eta^j(t)$ as the number of elements of the subtype j (potential hosts) that are present at the time point t. By including the biological fact that elements of a subtype j «appear» (first evolve and than distribute) at time points $\tau_1^j, \tau_2^j, ..., \tau_{n_j}^j$, we define $\eta^j(t)$:

$$\eta^j(t) = \begin{vmatrix} 0, \ t \le \tau_1^j \\ \dots \\ k, \ \tau_k^j < t \le \tau_{k+1}^j \\ \dots \\ n_j \ \ \tau_{n_j}^j < t \end{vmatrix} . \quad (1)$$

Thus the ratio:

$$F^j(t) = \frac{\eta^j(t)}{n_j} \ , \qquad (2)$$

can be considered as an empirical function of a random variable τ^j.

The probability $p_{i,j}(t)$ is considered to be proportional to $\eta^j(t)$:

$$p_{i,j}(t) = \alpha \cdot \eta^j(t) \text{ under the condition } t \in T_i. \qquad (3)$$

Further, we assume that $\alpha \cdot n_j << 1$. For the *i*-th series at $n_i \to \infty$ the Poisson distribution take place:

$$\mathrm{P}(\bigcap_{j=1}^{N}\{\mu_{i,j} = x_j\}) = \prod_{j=1}^{N} \frac{a_{i,j}^{x_j}}{x_j!} \cdot e^{-a_{i,j}} \ , \qquad (4)$$

where

$$a_{i,j} = \sum_{t \in T_i} p_{i,j}(t) = \sum_{k=1}^{n_i} p_{i,j}(\tau_k^i) . \qquad (5)$$

Accordingly, for all N series

$$\mathrm{P}(\bigcap_{i=1}^{N} \bigcap_{i,j}\{\mu_{i,j} = x_{i,j}\}) = \prod_{i=1}^{N} \prod_{i,j} \frac{a_{i,j}^{x_{i,j}}}{x_{i,j}!} \cdot e^{-a_{i,j}} \ . \qquad (6)$$

According to equations (5) and (3) we define α*ij* as

$$a_{i,j} = \alpha \cdot \sum_{k=1}^{n_i} \eta^j(\tau_k^i) . \qquad (7)$$

The last sum can be written as the Stieltjes integral:

$$a_{i,j} = \alpha \cdot \int_{-\infty}^{\infty} \eta^j(t) \cdot d\eta^i(t) \ , \qquad (8)$$

If we insert this in equation (2), we get the following:

$$a_{i,j} = \alpha \cdot n_i \cdot n_j \cdot \int_{-\infty}^{\infty} F^j(t) \cdot dF^i(t)\ . \qquad (9)$$

As an approximation for $F^j(t)$ we chose the normal distribution with the parameters t_j, σ_j

$$F^j(t) = F\left(\frac{t - t_j}{\sigma_j}\right), \qquad (10)$$

where $F(x) = \frac{1}{\sqrt{2\pi}} \int_{-\infty}^{x} e^{-\frac{y^2}{2}} dy$ - is the standard function of the normal distribution.

Then, within (9), we receive

$$a_{i,j} = \alpha \cdot n_i \cdot n_j \cdot F_{i,j}\ , \qquad (11)$$

where

$$F_{i,j} = F\left(\frac{t_i - t_j}{\sqrt{\sigma_i^2 + \sigma_j^2}}\right). \qquad (12)$$

Thus, the problem is reduced to the search for estimations of the unknown parameters α, $t_1 \ldots t_N$, $\sigma_1 \ldots \sigma_N$.

To estimate these parameters we use a maximal likelihood method. Replacing in (6) $x_{i,j}$ with $m_{i,j}$ and lowering the multipliers not dependent on estimated parameters, and in view of (11), we receive the function of likelihood

$$l = l(\alpha, t_1 \ldots t_N, \sigma_1 \ldots \sigma_N) = \prod_{i=1}^{N} \prod_{j=1}^{N} \left(\alpha \cdot F_{i,j}\right)^{m_{i,j}} \cdot e^{-\alpha \cdot n_i \cdot n_j \cdot F_{i,j}}\ . \qquad (13)$$

Accordingly, the logarithm of function of likelihood is equal to

$$\ln(l) = \sum_{i=1}^{N} \sum_{j} \left(m_{i,j} \cdot \ln(\alpha \cdot F_{i,j}) - \alpha \cdot n_i \cdot n_j \cdot F_{i,j}\right). \qquad (14)$$

Combining $F(-x) = 1 - F(x)$ and (12) we get $F_{i,j} + F_{j,i} = 1$ and $F_{i,i} = 1/2$.

So the sum:

$$n_0 = \sum_{i=1}^{N} \sum_{j=1}^{N} n_i \cdot n_j \cdot F_{i,j} + \sum_{i=1}^{N} n_i \cdot n_i \cdot F_{i,i} = \sum_{i=1}^{N-1} \sum_{j=i+1}^{N} n_i \cdot n_j + 1/2 \sum_{i=1}^{N} n_i^{\ 2} , \tag{15}$$

does not depend on any of the parameters. We also suppose:

$$m_0 = \sum_{i=1}^{N} \sum_{j=1}^{N} m_{i,j} , \tag{16}$$

It is then possible to describe (14) in the form of

$$\ln(l) = \sum_{i=1}^{N} \sum_{j=1}^{N} m_{i,j} \cdot \ln(F_{i,j}) + m_0 \cdot \ln(\alpha) - n_0 \cdot \alpha . \tag{17}$$

Equating zero to a partial derivative

$$\frac{\partial \ln(l)}{\partial \alpha} = \frac{m_0}{\alpha} - n_0 ,$$

we receive an estimation for α:

$$\tilde{\alpha} = \frac{m_0}{n_0} \tag{18}$$

$F_{i,j}$ do not change under the replacement:

$$\begin{array}{l} \sigma_i \to h \cdot \sigma_i \\ t_i \to h \cdot t_i + t_0 \end{array} .$$

Therefore, it is possible to limit t_i to the condition $\sum_{i=1}^{N} t_i = 0$.

Concerning parameter σ_i we have accepted they are proportional (in particular $\sigma_i = n_i$).
