# Supplementary material for: A novel web-based TinT application and the chronology of the Primate Alu retroposon activity
Source: BMC Evol Biol. 2010 Dec 2;10:376. doi: 10.1186/1471-2148-10-376 (PMC3014933; doi:10.1186/1471-2148-10-376)

Additional file 2: **TinT tutorial**

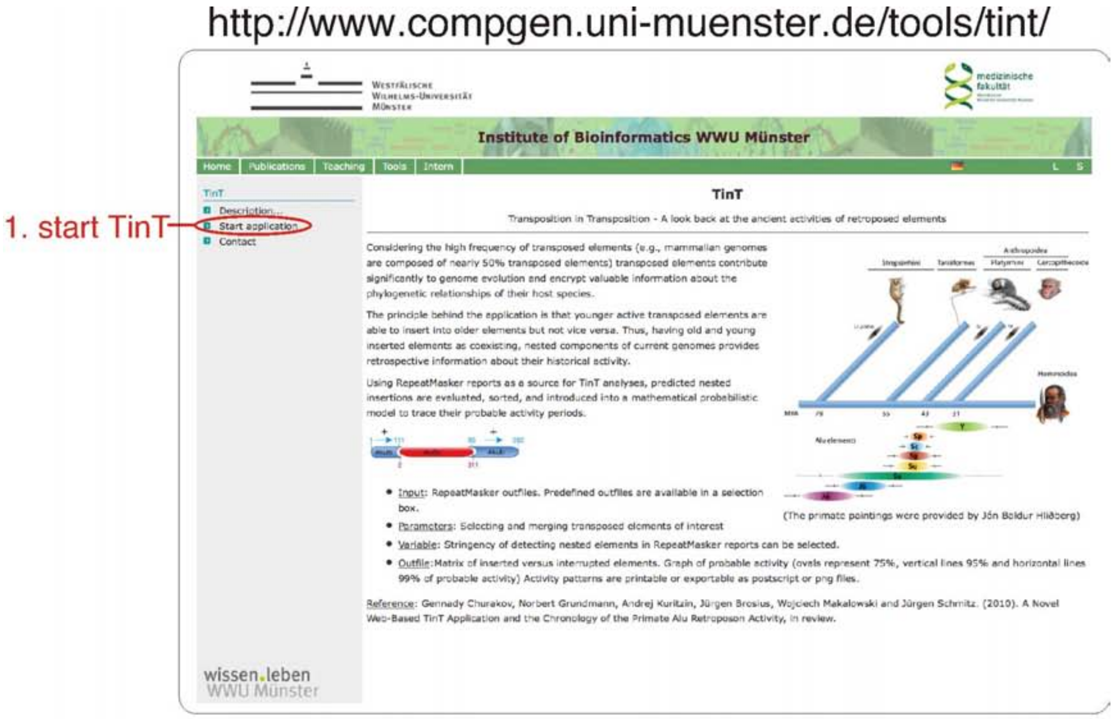

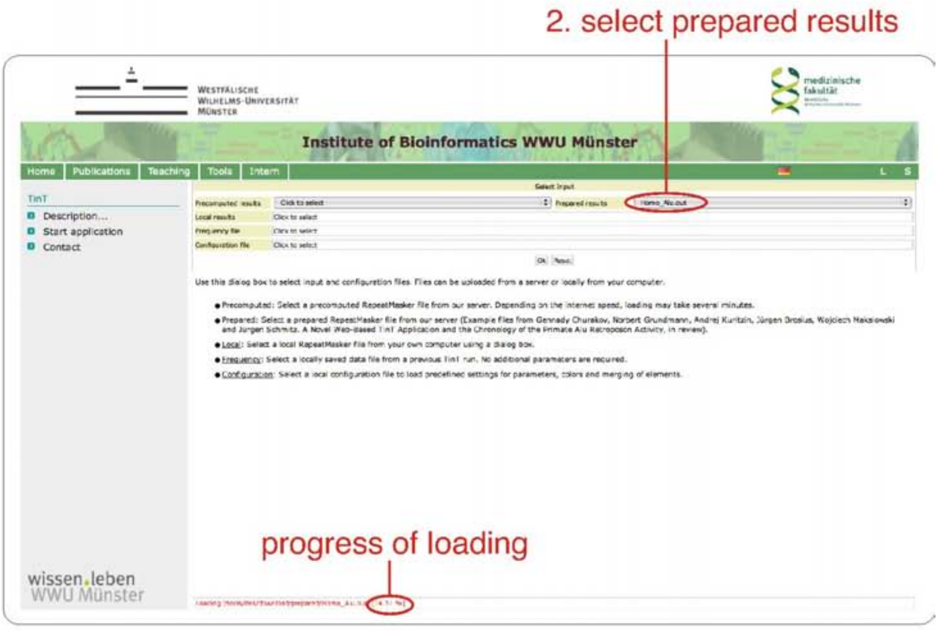

alternatively you can select precomputet results (genomes)
or local results (your own RepeatMasker.out file)
or frequency files (previously saved projects)
and load presettings (configurations from previously saved projects)

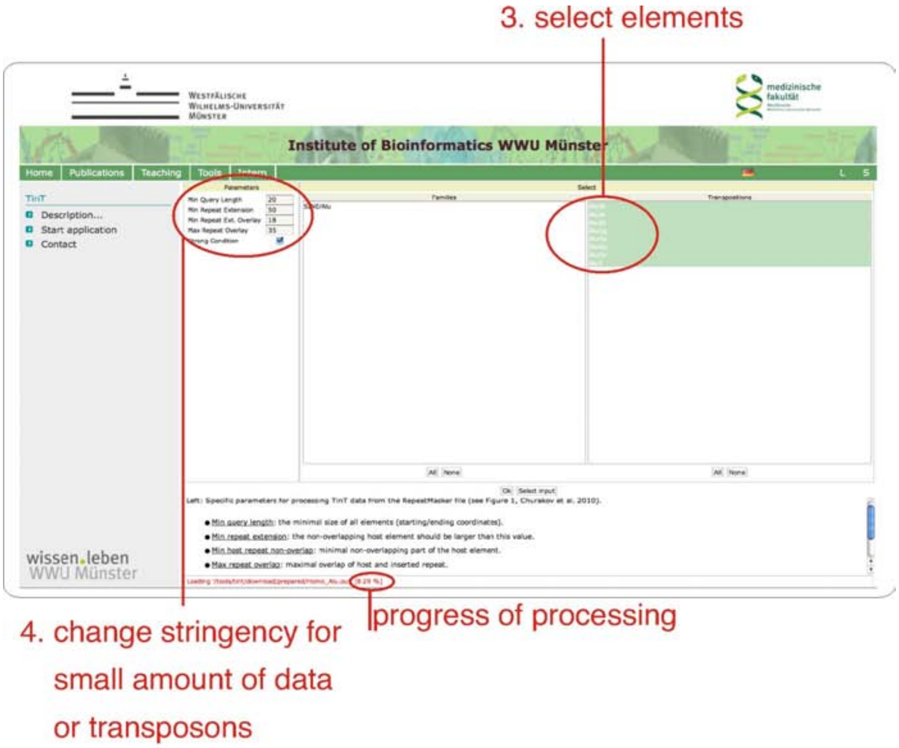
3. select elements
Westfälische Wilhelms-Universität Münster
medizinische fakultät
Institute of Bioinformatics WWU Münster
Home
Publications
Teaching
Tools
Intern
TinT
Description...
Start application
Contact
wissen.leben
WWU Münster
4. change stringency for small amount of data or transposons
progress of processing

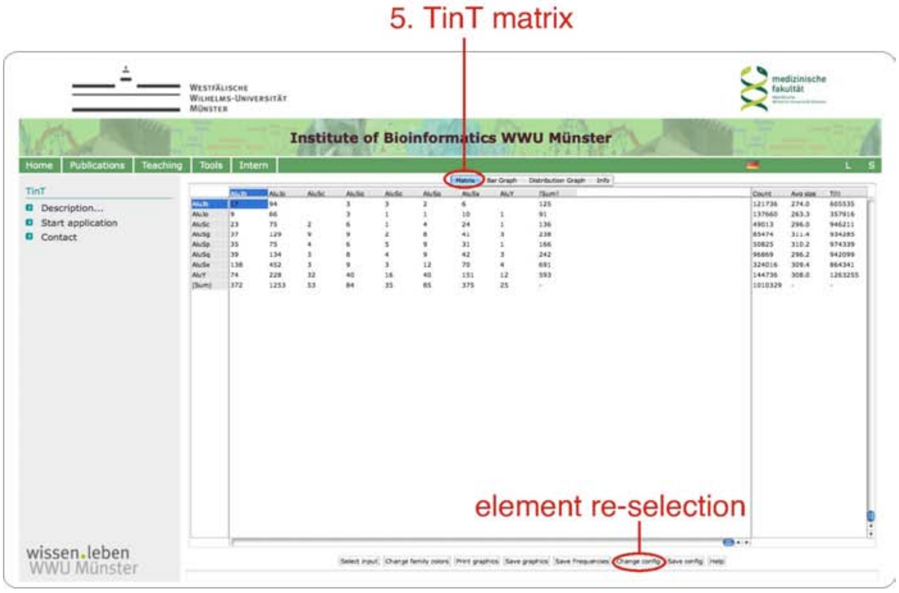
5. TinT matrix
Westfälische Wilhelms-Universität Münster
medizinische fakultät
Institute of Bioinformatics WWU Münster
Home
Publications
Teaching
Tools
Intern
TinT
Description...
Start application
Contact
element re-selection
wissen.leben
WWU Münster

6. bar graph

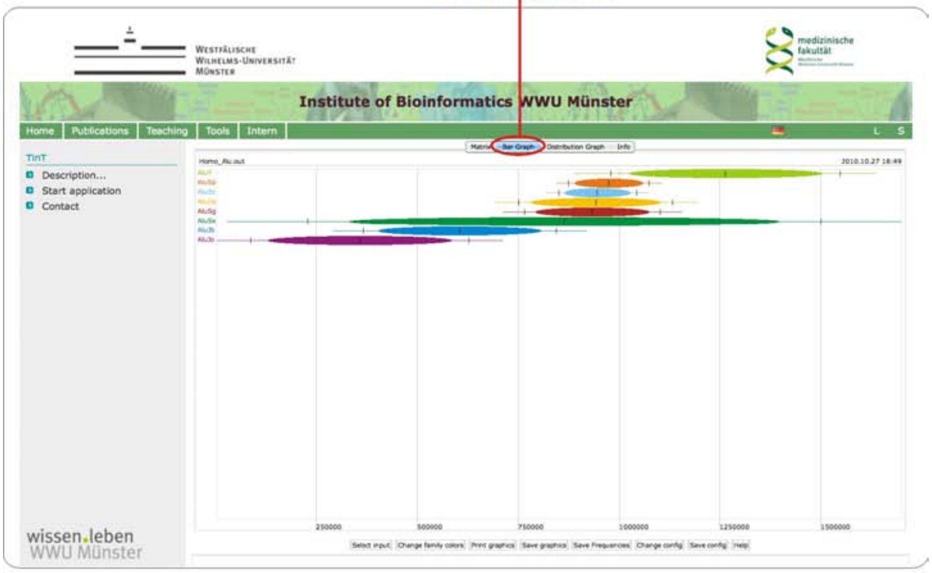

7. distribution graph

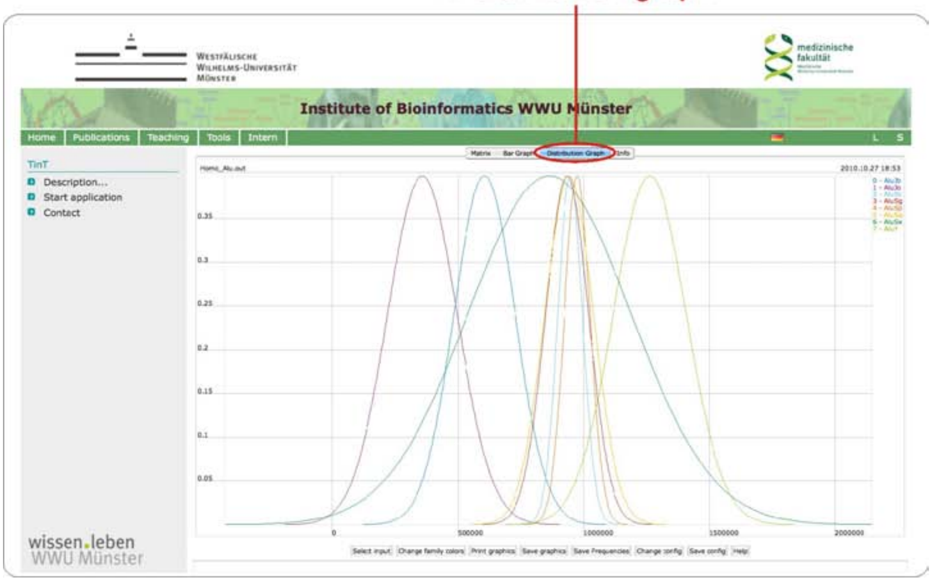

Supplement: Additional file 2 — TinT tutorial [file 1471-2148-10-376-S2.PDF]
