# Supplementary material for: A novel web-based TinT application and the chronology of the Primate Alu retroposon activity
Source: BMC Evol Biol. 2010 Dec 2;10:376. doi: 10.1186/1471-2148-10-376 (PMC3014933; doi:10.1186/1471-2148-10-376)
Supplement: Additional file 3 — Sequence-based phylogeny of Alu elements [file 1471-2148-10-376-S3.PDF]

Additional file 3: **Sequence-based phylogeny of *Alu* elements**

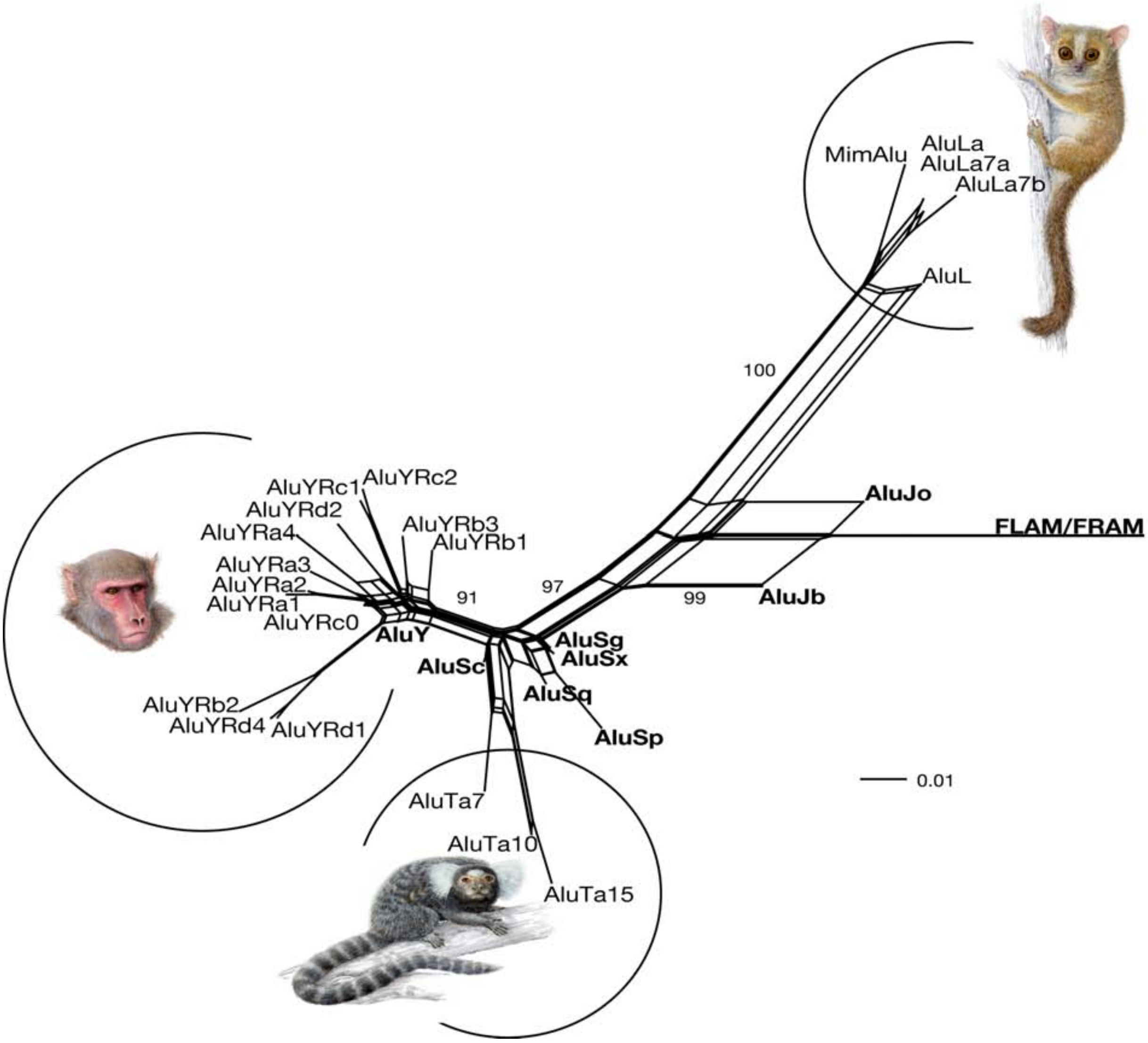

Additional file 3: SplitsTree4 (V4.10; Huson and Bryant 2006) implements a split decomposition method and was used to reconstruct the phylogenetic network of dimeric *Alu*. Networks display more realistic evolutionary scenarios, visualizing noise as parallelograms, compared to a strict tree-like topology (ideal data will form a distinct tree topology, noisy data will give rise to a tree-like network). As an example, the long edge separating *Alu*Jo and *Alu*Jb

indicates the close relatedness of *Alu*Jo to the lemur (strepsirrhines)-specific elements. Competitively, the long edge separating *Alu*Jo and *Alu*Jb from all other elements reflects signals of their relatedness. We used the NeighborNet- and LogDet- distances to derive the evolutionary network (30 “taxa”, 361 characters). Tree drawing was performed with the EqualAngle method. Bootstrap estimation was performed with 10,000 iterations to evaluate the main splits between *Alu*J, *Alu*S, and *Alu*Y elements. 0.01 denotes number of nucleotide substitutions per site.

The lemur-specific elements (*Alu*L, *Alu*La, *Alu*Mim) are shown to be most closely related to the *Alu*Jo elements, the only other dimeric *Alu* present in the grey mouse lemur. The New World marmoset-specific *Alu*Ta elements are derived from an *Alu*S progenitor. The rhesus monkey specific *Alu*R elements are most closely related to the *Alu*Y elements. The significant separations between the *Alu*J, *Alu*S and *Alu*Y elements are well supported (bootstrap 91-100), indicating clearly resolved relationships; however, the relationships within the *Alu*S subfamily are not significantly resolved. As shown by the TinT distributions, the various *Alu*S elements were active during the same period and they did not leave behind significant phylogenetic signals to clearly resolve their more detailed affiliations using these methods. The location of the root is indicated by FLAM/FRAM, the proposed progenitors of *Alu* dimeric elements.

Reference

Huson DH, Bryant D: **Application of phylogenetic networks in evolutionary studies.** *Mol Biol Evol* 2006. **23**: 254-267.
